# Fast method for assessing binder migration in lithium-ion battery electrodes using glow discharge-sector field-mass spectrometry

**DOI:** 10.1038/s42004-026-02210-4

**Published:** 2026-09-22

**Authors:** S. L. Dorn, J. Kauling, M. Börner, M. Winter, S. Wiemers-Meyer, S. Nowak

**Affiliations:** 1 University of Münster, MEET Battery Research Center, Münster, Germany; 2 Helmholtz-Institute Münster, IMD-4, Forschungszentrum Jülich GmbH, Münster, Germany

**Keywords:** Materials for energy and catalysis, Mass spectrometry

## Abstract

A high amount of energy needed for the production of lithium-ion batteries is used for the drying and solvent recovery of *N*-methyl-2-pyrrolidone (NMP) in electrodes with polyvinylidene fluoride-based binder (PVdF) systems. Therefore, controlling the amount of used solvent as well as developing efficient drying procedures is of major importance. Furthermore, the drying process and solid content of the electrode paste directly affect the battery performance due to the risk of transport of the binder towards the electrode surface. This work focuses on an analytical approach for rapidly and reliably assessing the degree of this migration by measuring the fluorine distribution *via* glow discharge-sector field-mass spectrometry (GD-SF-MS). A sample with notably more binder migration based on preliminary cross-section imaging *via* scanning electron microscopy combined with energy-dispersive X-ray spectroscopy and a reference sample with no visualized binder migration were evaluated, with the former displaying a severely elevated degree of binder migration over the reference based on GD-SF-MS analysis. The developed method could also be applicable to electrodes based on sodium carboxymethyl cellulose (Na-CMC) binders by investigating the Na distribution instead.

## Abstract (web summary)

The performance of lithium-ion batteries is influenced by the electrode drying process due to the risk of binder transport, highlighting the need for efficient methods for assessing binder migration. Here, the authors propose an approach for assessing the degree of such migration based on ion distribution measurements using glow discharge-sector field-mass spectrometry that could be applied to binder systems based on polyvinylidene fluoride or sodium carboxymethyl cellulose.

## Introduction

One of the most important challenges the world faces for controlling the man-made climate change is the reduction carbon emissions by utilizing inherently fluctuating renewable energy sources instead of fossil fuels^1–3^. This entails the need for reliable energy storage technologies such as lithium-ion batteries (LIBs)^3–5^. First introduced commercially by Sony in 1991^6–8^, demand for them has increased radically ever since^9,10^, which is accompanied by an increasing energy demand for LIB manufacturing^11–14^. Roughly a third to half of all energy used in the production of LIBs can be attributed to the drying and solvent recovery in cathode manufacturing utilizing *N*-methyl-2-pyrrolodine (NMP) as the solvent in polyvinylidene fluoride (PVdF) binder systems^12,14–16^. It is therefore vital to optimize the solid content of the electrode paste as well as the drying procedure during processing to reduce the overall energy demand in LIB manufacturing. However, there are some limitations and challenges, as both the drying procedure and the amount of solvent used can directly affect the structural integrity and thus electrochemical performance of the electrodes due to a phenomenon called binder migration^17–19^. Binder migration, which is defined as the transport of binder material towards the electrode surface, is increasingly recognized as a critical factor governing electrode integrity and performance^20^. During the drying procedure in wet processing, capillary-driven convection and solvent evaporation can drive the PVdF binder and conductive agent (CA) (collectively referred to as carbon-binder domain, CBD) towards the outer regions of the electrode^17^. This non-uniform distribution detrimentally affects the particle-particle and particle-current collector contact, leading to an increased electronic resistance, reduced adhesive strength and accelerated capacity fade^19,21^. Therefore, the impact of different electrode paste formulations and drying procedures on the electrode need to be monitored and assessed continuously to ensure that any binder migration can be held to a minimum^22^.

A recent study using pyrolysis gas chromatography hyphenated to mass spectrometry (Py-GC-MS) and cross-sectional secondary electron microscopy (SEM) combined with energy-dispersive X-ray spectroscopy (EDX) visualized depth-dependent PVdF distributions in cathodes and correlated these gradients with drying temperatures^17^. Another study utilized the highly toxic and volatile osmium tetroxide (OsO_4_) to stain the SBR binder fractions in negative electrodes and subsequently analyze the binder fractions *via* resin-embedded imaging^23^. Similarly, staining of SBR was also conducted recently utilizing silver nitride (AgNO_3_) and bromine (Br_2_)^24^.

Additionally, X-ray photoelectron spectroscopy (XPS) as a common surface sensitive technique in the field of LIBs struggles with several limitations for assessing binder migration. XPS only probes the outermost few nanometers of the electrode, preventing the evaluation of the binder distribution across the upper electrode layers, while argon (Ar) sputter depth profiling introduces polymer artifacts, making analysis of binder components unreliable^25,26^.

Further drawbacks to the above methods is that they are time consuming, unreliable, dangerous and require extensive sample preparation, which makes rapid and routine analysis parallel to electrode manufacturing unfeasible. Another analytical technique that can alleviate these shortcomings is glow discharge-sector field-mass spectrometry (GD-SF-MS). GD-SF-MS can analyze solid samples such as LIB electrodes directly by sputtering the sample *via* an Ar plasma and delivering an elemental depth-profile over several µm^27–30^. This technique offers several excellent qualities such as fast measurement times of as little as a few minutes, high and low sputter rates due to the possibility of a pulsed operation mode and a large range of measurable concentrations due to the triple detection mode of the sector field consisting of a Faraday cup and a secondary electron mulitplier^31,32^. Additionally, since solid samples can be analyzed directly, sample preparation is minimal for mechanically stable samples such as LIB electrodes. The different ionization mechanisms occurring in the GD ion source further allow for the ionization of fluorine (F), which is generally not possible for common plasma-based ionization methods such as inductively coupled plasmas (ICP) due to the higher excitation energy of F^+^ compared to Ar^+^^33^, which would typically lead to a preferred ionization of Ar in the plasma over F. Since ionization occurs in the plasma’s afterglow, F may instead be ionized *via* high energy electron impact rather than thermal excitation or charge exchange as is the case in ICP-based ionization^34^. The lower Ar gas flow in GD systems (300–500 mL min^−^^1^) compared to common ICP systems (10 to 20 L min^−^^1^) further reduces the probability of a charge exchange between an ionized F^+^ and an Ar atom in the plasma. This allows for the reliable analysis of F *via* GD-SF-MS^35–37^, as the medium and high resolutions of the SF-MS ( ≈ 4000 and ≈ 10,000 respectively) are sufficient to separate the ^19^F peak from *e.g.*, water-based ion interferences in the Ar supply (^18^O^1^H^+^, ^16^O^1^H_3_^+^, ^17^O^2^H^+^, ^16^O^1^H_2_^2^H^+^ etc.).

In the context of this work, GD-SF-MS can serve as a powerful analytical method for assessing the PVdF distribution in electrode samples based on the F signal. This study examines two different NCM622 (LiNi_0.6_Co_0.2_Mn_0.2_O_2_, lithium nickel (Ni) cobalt (Co) manganese (Mn) oxide) cathode formulations, reference samples (ref. samples) and samples with notable binder migration (samples A - C) based on preliminary SEM-EDX investigations. The samples were then analyzed *via* GD-SF-MS to acquire depth-resolved F signals to evaluate whether differences in the degree of binder migration can be identified reliably.

## Results and discussion

### Preliminary cross-section imaging investigation

**Fig. 1:**
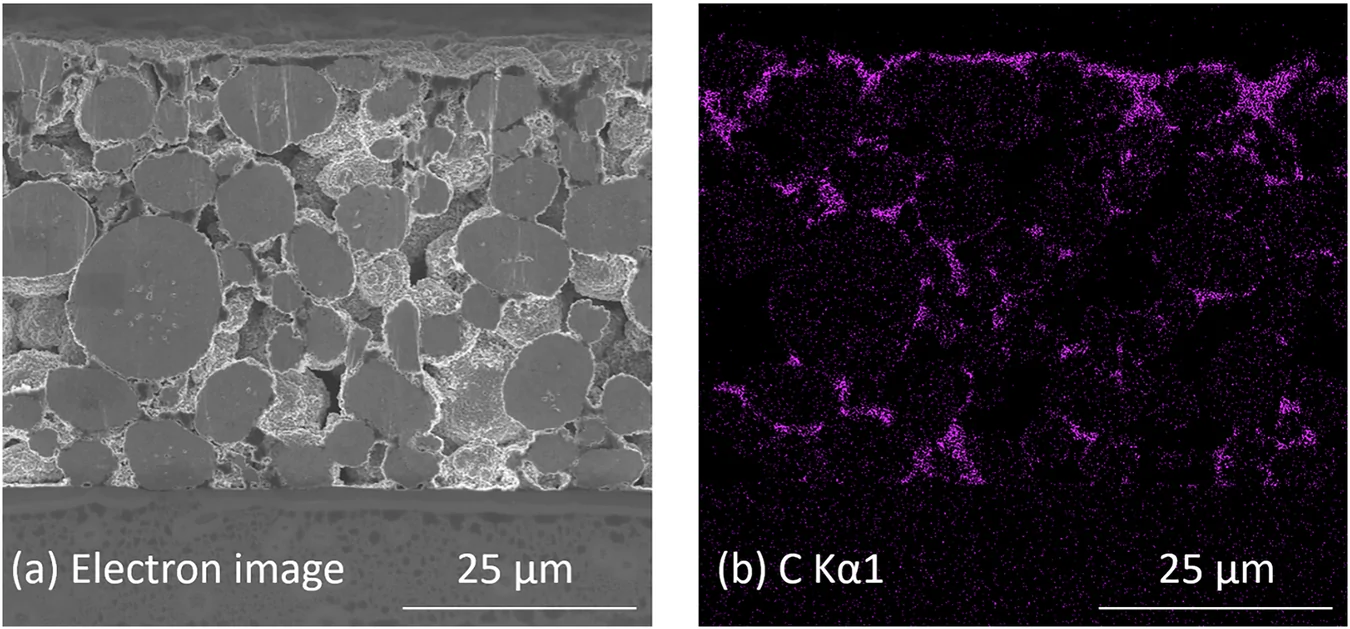
SEM/EDX cross-section imaging of an NCM622 sample with CDB accumulation at the surface. Panel (**a**) depicts an electron image using SEM, while panel (**b**) depicts the carbon signal using EDX imaging.

SEM and EDX imaging for the cross-section of a cathode sample displaying pronounced binder migration can be seen in Fig. 1. The SEM image (Fig. 1a) depicts the polished surface of the cross-section with densely packed NCM622 particles with typical polycrystalline morphology. Interstitial regions between the active material particles are filled with a darker-shaded matrix, which can be attributed to the amorphous CBD. The sample further displays a non-uniform porosity distribution, with larger voids visible in the upper regions of the electrode. The corresponding C Kα1 map (Fig. 1b) depicts a heterogeneous distribution of C throughout the electrode’s thickness. A pronounced increase can be seen towards the upper regions of the electrode, indicating an enrichment of carbon-containing species such as the binder itself and the CA. Within the bulk of the electrode, the C signal is localized along the interparticle boundaries. Given that both the PVdF binder as well as the CA contain carbon, it can be concluded that the accumulation of it towards the surface of the electrode can be attributed to binder migration during processing.

### GD-SF-MS depth profiling

**Fig. 2:**
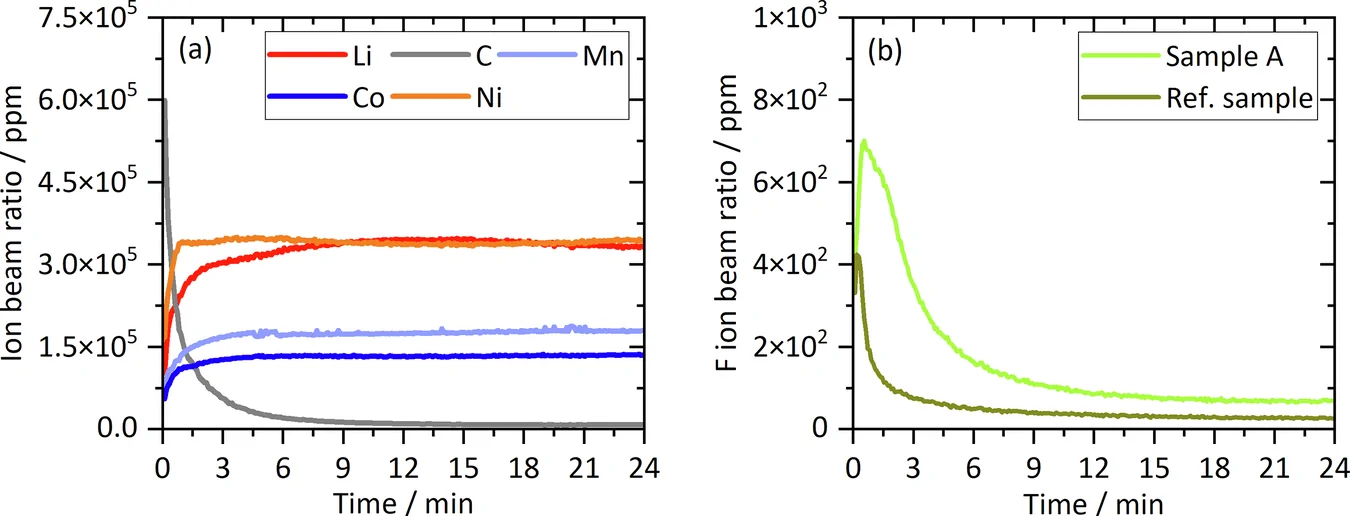
GD-SF-MS depth-profiling of two NCM622 cathode samples. Panel (**a**) depicts the elemental depth profile of major elements (Li, C, Mn, Co, Ni) from sample A with notable binder migration *via* their calculated ion beam ratios (IBRs). Panel (**b**) depicts the respective F IBRs for both the sample A as well as the reference sample.

After the identification of the CBD distribution *via* cross-section imaging, GD-SF-MS analysis was conducted for a reference sample as well as the identified binder migration sample A. Exemplary results for the elemental distribution of Li, C, Mn, Ni and Co in sample A can be seen in Fig. 2a.

The ion beam ratio (IBR) is depicted on the y-axis and represents the relative signal intensity of an element compared to the sum of measured intensities of all elements and serves as a parameter that excludes plasma or excitation instabilities within a measurement^29,38,39^. The IBRs are calculated according to Equation S1 in the SI.

The measurement reveals a notable increase of the C IBR at the surface of the electrode with up to 6.0·10^5^ ppm. This drops down to a stable 0.8·10^5^ ppm after around 15 min of sputtering, indicating that the bulk of the electrode is fully reached by this time. Correspondingly, the IBRs of Li and the transition metals begin at lower values at the start of the measurement. These IBRs increase sharply over the first 2–3 min of sputtering, reaching stable values after 9–12 min. This behavior can be explained by accumulation of carbon containing species at the surface of the electrode, which aligns well with results obtained *via* SEM-EDX imaging. Carbon contamination at the sample surface is a common occurrence in sensitive surface analysis^40–43^, as any organic contamination and the adsorption of gases such as carbon dioxide or other organic volatile compounds at the sample surface directly impact the carbon signal obtained with these measurements. Additionally, other carbon containing compounds such as carbonates originating from the sample itself (*e.g.*, residual carbonate species from the active material precursors) can further influence the carbon signal obtained. The increased C IBR therefore does not exclusively represent the migration of the carbon containing binder and CA, which is why the F distribution was investigated more closely and in direct comparison with the results for the reference sample in Fig. 2b.

Here the IBRs show a similar profile to the C IBRs obtained for sample A. While the measurement starts out with relatively lower F contents during the first few seconds for both sample A as well as the reference sample (421 ppm and 331 ppm respectively), a sharp increase to a local maximum can be identified for both samples as well, reaching values of up to 701 ppm for the sample A and 424 ppm for the reference sample, which then fade into a stable bulk content after around 15 min. These results indicate that the reference sample also depicts a detectable level of binder migration, since even a controlled evaporation of solvent would naturally lead to a certain amount of binder being dragged towards the surface of the electrode through capillary-driven convection during the drying process^17^. Regardless, in addition to the overall higher F IBRs obtained for sample A, the results still present a qualitative difference in the F distribution between the two samples. Sample A depicts a notably broader F distribution at the surface compared to the reference sample, which may be attributed to a more pronounced accumulation of the PVdF binder at the surface.

**Fig. 3:**
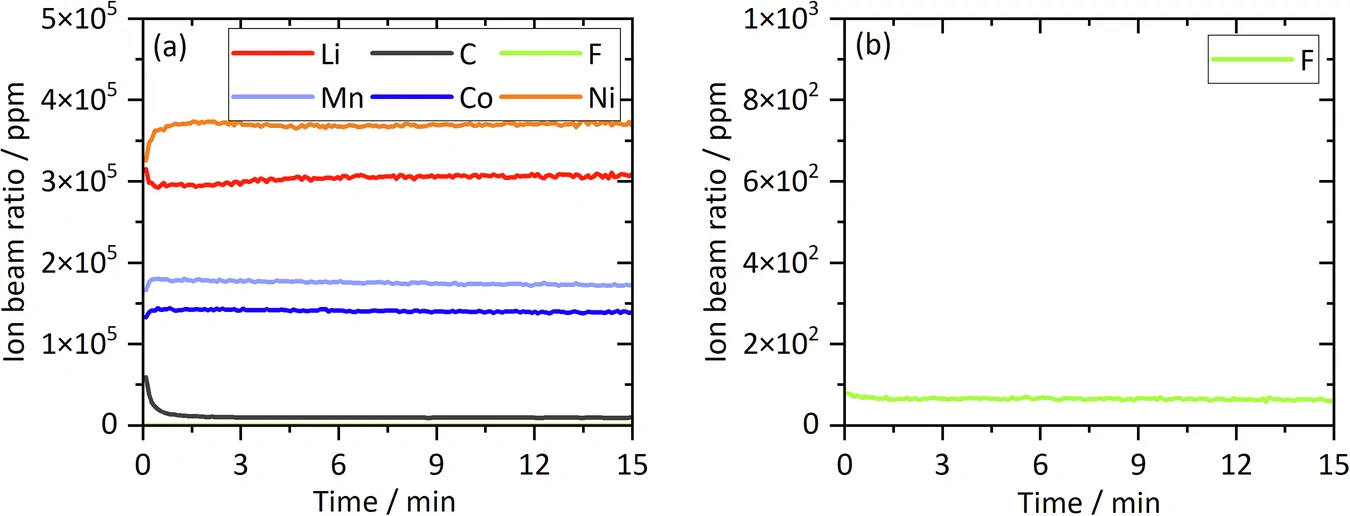
GD-SF-MS plasma ignition check *via* a second analysis after initial sputtering, subsequent removal and storage at normal atmosphere for one day. Panel (**a**) depicts the elemental depth profile of all measured elements *via* their calculated IBRs, panel (**b**) depicts the zoomed in IBRs for F for visibility purposes.

A plasma ignition check was performed further to ensure that the gradients at the surface were not influenced by the plasma ignition causing a change in the initial excitation behavior during analysis, which could potentially result in the preferred excitation of the organic CBD over the inorganic oxides of the electrode’s active material. This means that after sample A was measured initially, it was removed from the instrument and kept at normal atmosphere for one day. Afterwards, a second measurement of the same sample on the already sputtered area was conducted. The results of that measurement can be seen in Fig. 3.

Overall, the IBRs for all elements show stable signals throughout the entire measurement due to already having reached the bulk material in the initial measurement void of any binder accumulation (Fig. 3a). The exception is a short deviation during the first few seconds of analysis. During this, an elevation of the Li and C IBRs can be observed which causes a decrease in the IBRs of the transition metals and leaves the F IBR unaffected (Fig. 3b). This can likely be attributed to the formation of lithium carbonate on the surface of the already sputtered material due to the increased reactivity of the freshly exposed material surface against the moisture in the atmosphere. Since this effect appears to be notably smaller than the concentration gradients that could be observed during the initial measurement, it can be concluded that the ignition of the plasma itself has no impact on the excitation behavior at the start of the measurement. Thus, it can be excluded as a contributing factor to the F gradient that can be observed in the samples.

### Normalization and comparison of F fractions

**Fig. 4:**
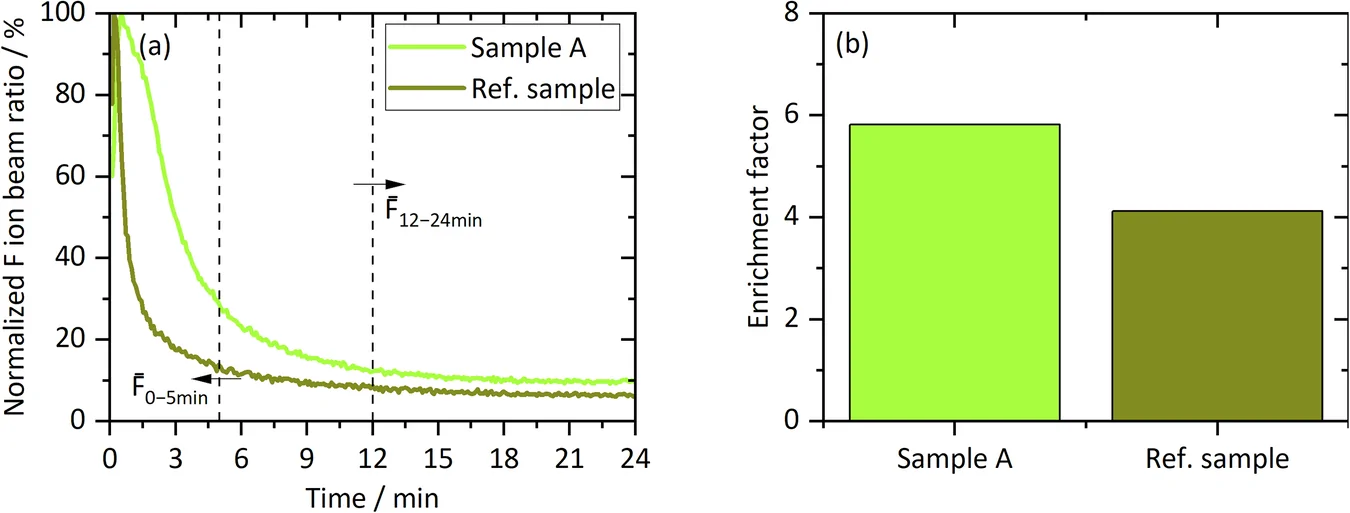
Normalization and integration of F IBRs from GD-SF-MS analysis. Panel (**a**) depicts F IBRs for the reference NCM622 sample and sample A normalized to the respective maximum IBR with marked cutoff regions for enriched surface *vs.* electrode bulk. Panel (**b**) depicts the corresponding calculated enrichment factor as a ratio of F̅_0-5 min_ to F̅_12-24 min_.

For a more adequate direct comparison between the degrees of binder migration in the different samples, a normalization of the F IBRs for the respective analyses is necessary. The objective of this comparison is not to quantify the total fluorine content of the analyzed regions – since the GD-SF-MS results are not quantitative without external calibration – but to evaluate the relative redistribution of the F content within the electrode. Normalization to the maximum F IBRs preserves the relative decrease of the fluorine signal from the surface layers compared to the electrode bulk and can be utilized to highlight the differences in the F enrichment profiles in the different samples. Additionally, an enrichment factor (EF) was determined as the ratio of the average F content over the first 5 minutes of analysis (F̅_0-5 min_) as a representation of the F content in the enriched surface layer *vs*. the last half of analysis (F̅_12-24 min_) as a representation of the electrode bulk. These results can be seen in Fig. 4.

While the normalization eliminates the higher F IBRs in sample A, the overall broader peak of the F signal persists (Fig. 4a). This can be attributed to an increased accumulation of the binder at the surface of the electrode compared to the reference sample due to more binder migrating towards the upper regions during the drying process. This is also reflected in the EF values. The EF for sample A, with more pronounced binder migration, amounts to 5.8, and the EF for the reference sample to 4.1. This represents an increase of 41% over the reference sample, which means that the degree of binder migration occurring in the respective cathodes can be considered 41% more severe in sample A compared to the reference.

## Conclusion

Based on preliminary SEM-EDX, this study could show that sample A with CBD accumulation in the upper electrode layers displayed a notably higher level of binder migration compared to the reference sample based on the investigated F distribution. However, according to results obtained *via* GD-SF-MS, even the reference sample displayed a low level of binder migration that may be challenging to detect with more conventional analysis methods such as SEM-EDX cross-section imaging. This can be attributed to the GD system’s unique ability to ionize and detect F, which makes it more reliable and sensitive at identifying binder fractions in the electrode regions compared to using the carbon signal.

Overall, GD-SF-MS can serve as a potent tool in conducting rapid analysis of electrodes to identify binder migration. In addition to investigating F-based binder systems, which was shown in this work, other systems utilizing e.g., Na-CMC should also be assessable. The analysis requires minimal sample preparation and achieves reliable and reproducible results (see Fig. S1 in the SI) in as little as 15 min, which is crucial for assessing and monitoring binder migration parallel to optimization of the drying process as part of manufacturing.

## Methods

### Electrode and sample preparation

Electrodes were formulated using a ratio of 95/2/3 wt.% of NCM622 (BASF, Ludwigshafen, Germany)/Super C65 as CA (Imerys Graphite & Carbon, Paris, France)/PVdF (Sigma Aldrich, Burlington, MA, USA). The solid content of the reference electrode paste was set to 80%, the solid content of the electrodes displaying elevated binder migration was set to 73%. After dissolving the binder in NMP (Sigma Aldrich, Burlington, MA, USA), prepared dry mixtures of the NCM622 and CA were added and dispersed for 40 min at 4000 rpm (Dispermat CV3-Plus, VMA-Getzmann, Reichshof, Germany). The resulting paste was coated on aluminum foil using a doctor blade, and oven-drying was conducted for 2 h at 80 °C. The electrode sheets were then calendered to a porosity of 30%.

Electrodes for GD-SF-MS analysis were punched into circular samples with a diameter of 12 mm using a handheld puncher. Cross-section preparation of samples was conducted using an IB-19540CCP cooling cross-section polisher (Jeol, Akishima, Tokyo, Japan) with an acceleration voltage of 5 kV and an additional smoothing step at 2 kV.

### Scanning electron microscopy and energy-dispersive X-ray spectroscopy

SEM imaging was conducted using an Auriga CrossBeam 550 workstation (Zeiss, Oberkochen, Germany) equipped with a Schottky-type field emission gun and an acceleration voltage of 3 kV. EDX imaging was conducted using an Ultim Extreme detector (Oxford Instruments, High Wycombe, England) with an acceleration voltage of 15 kV. Evaluation was conducted using the AZtech software (Oxford Instruments, High Wycombe, England).

### Glow discharge-sector field-mass spectrometry

GD-SF-MS analysis in pulsed-mode was conducted using a Thermo Element GD Plus (Thermo Scientific, Bremen, Germany). Measurements of samples with notable binder migration and reference samples were conducted three times, representative data of one measurement for each sample is shown in this study. The prepared electrode samples were attached to a brass sample holder using a double-sided adhesive pad (Plano, Wetzlar, Germany) and inserted into the instrument. For the plasma ignition check, the sample was removed after initial measurement, stored at normal atmosphere for one day and subsequently analyzed a second time. Analysis parameters were applied according to Table 1. Masses for all detected elements (^7^Li, ^12^C, ^19^F, ^55^Mn, ^58^Ni, ^59^Co) were acquired at medium resolution ( ≈ 4000). The resulting crater depth of each analysis is estimated to be around 10 ± 5 µm based on reported crater depths in similar samples.

**Table 1:** Overview of the applied instrument parameters used for GD-SF-MS analysis

Plasma voltage	1000 V
Plasma current	8 mA
Ar gas flow	450 mL min^−^^1^
Pulse duration	10 μs
Pulse frequency	4 kHz
Cooling temperature	0 °C
Anode cap size	8 mm

## Supplementary information

SI

## Data Availability

The authors declare that the data supporting the findings of this study are available within the paper and its Supplementary Information files. Should any raw data files be needed they are available from the corresponding author upon reasonable request.
