# Supplementary material for: Fast method for assessing binder migration in lithium-ion battery electrodes using glow discharge-sector field-mass spectrometry
Source: Commun Chem. 2026 Sep 22;9:303. doi: 10.1038/s42004-026-02210-4 (PMC13598097; doi:10.1038/s42004-026-02210-4)
Supplement: Supplementary file 1 [file 42004_2026_2210_MOESM1_ESM.pdf]

## Supporting Information

The ion beam ratio (IBR) in GD-SF-MS analysis for element A in the matrix M is calculated according to Supplementary Equation 1:

$$\mathrm{IBR}_{\mathrm{M}}^{\mathrm{A}} = \frac{\frac{I_{\mathrm{A}}}{f_{\mathrm{A}}}}{\sum_{\mathrm{i}} \frac{I_{\mathrm{M,i}}}{f_{\mathrm{M,i}}}} \qquad (1)$$

$I_{\mathrm{A}}$ describes the measured intensity of an isotope of element A with an isotopic abundance of $f_{\mathrm{A}}$. $I_{\mathrm{M,i}}$ represents the measured intensity of matrix element isotope M,i with the isotopic abundance $f_{\mathrm{M,i}}$.

Supplementary Figure 1 reveals the reproducibility of the GD-SF-MS analysis based on samples A – C with notable binder migration. The F IBRs display slight quantitative differences for the repeated measurements, which can be attributed to several factors that influence the excitation and detection behavior across separate measurements, such as the vacuum conditions, lens alignment, detector calibration, temperature, magnetic field in the sector-field or standard variation that can often occur in solid sampling. However, normalization can account for many of these factors and reveals practically identical gradients for all measurements of the same sample.

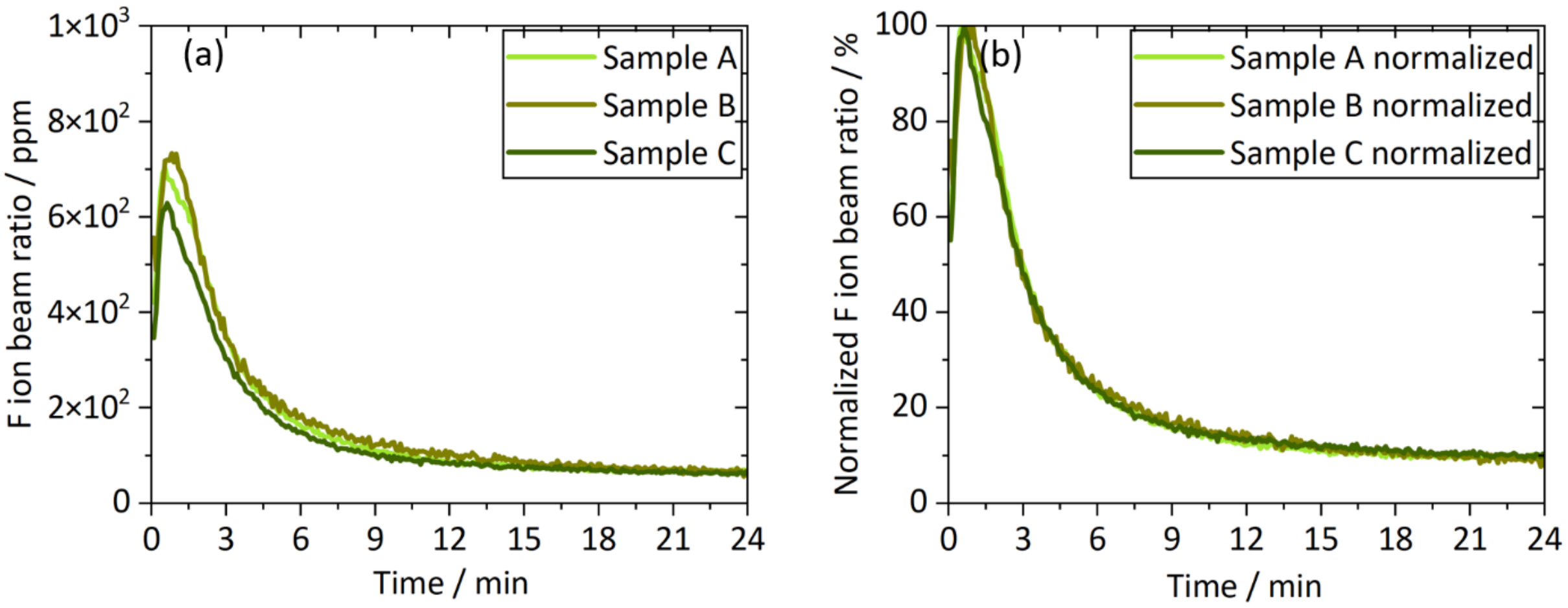


*Supplementary Figure 1: Results for repeated measurements of the NCM622 samples A – C depicting notable binder migration. Panel (a) depicts the F IBRs with slight quantitative differences, while panel (b) depicts the normalization of the F IBRs, revealing the high reproducibility of the qualitative assessment of the binder distribution.*
